# New-Onset and Persistent Insulin-Dependent Diabetes in Patients With COVID-19: A Peruvian Experience

**DOI:** 10.7759/cureus.27272

**Published:** 2022-07-26

**Authors:** Anthony Ramos-Yataco, Emanuel A Salcedo Davila, Kelly Meza, Inga Harbuz-Miller

**Affiliations:** 1 Internal Medicine, Hospital Ricardo Cruzado Rivarola, Nasca, PER; 2 Department of Medical Education, Ricardo Palma University, Lima, PER; 3 Internal Medicine, Hospital Nacional Guillermo Almenara, Lima, PER; 4 Department of Endocrinology, University of Rochester, Rochester, USA

**Keywords:** insulin, peru, dka, new-onset dia-betes, covid 19

## Abstract

Type 2 diabetes mellitus (T2DM) has been recognized as a risk factor for severe coronavirus disease 2019 (COVID-19) infection, and COVID-19 in diabetic patients is associated with a poor prognosis. New evidence suggests that patients with T2DM may experience diabetic ketoacidosis (DKA) or hyperglycemic hyperosmolar state (HHS) if infected with severe acute respiratory syndrome coronavirus 2 (SARS-CoV-2). However, there is limited literature on new-onset diabetes in patients infected by SARS-CoV-2 without a history of diabetes. We present a case series of three patients who developed new-onset diabetes while suffering from acute COVID-19 infection; they presented with DKA even though they had no prior history or risk factors for diabetes. They remain persistently insulin-dependent several months post-recovery.

## Introduction

The ongoing coronavirus disease 2019 (COVID-19) pandemic has affected more than 472 million people worldwide [1]. According to a recent report by the World Health Organization (WHO), the disease has led to more than six million deaths globally [1,2]. Of note, this pandemic has placed an enormous burden on Latin American countries, resulting in catastrophic outcomes such as the collapse of the already fragile healthcare systems, the scarcity of resources in hospital settings, high rates of community transmissions, and elevated mortality rates [3,4]. Recently, the Pan American Health Organization (PAHO) reported that COVID-19 has caused 2,770,509 deaths in the Region of the Americas [5]. One of the most affected countries in Latin America is Peru, which has reported a total of 213,685 deaths as of July 2022 [6]. This has been attributed to the country's fragmented healthcare system, ongoing economic crisis, and inadequate response and management strategies on the part of the government [3,6].

Although severe acute respiratory syndrome coronavirus 2 (SARS-CoV-2) infection has been primarily described as a respiratory infection, it has been demonstrated that it has the potential to affect a wide variety of organ systems. Of special interest is the endocrine system, where tissue dysregulation has been documented in many endocrine tissues such as the pancreatic, thyroid, and adrenal glands [7]. Also, metabolic complications of SARS-CoV-2, including DKA and HHS, have been described in patients with type 2 diabetes mellitus (T2DM) [8,9].

According to the Peruvian Center of Epidemiology, the prevalence of diabetes among patients hospitalized for COVID-19 is 10.9%, and it was as high as 12.3% in patients who had died of COVID-19 [10]. Nonetheless, there is scarce data on new-onset diabetes mellitus presenting as diabetic ketoacidosis (DKA) in the context of COVID-19. In this report, we discuss the cases of three patients with new-onset diabetes mellitus presenting with DKA while infected with SARS-CoV-2 who continue to experience persistent insulin dependence long after their recovery from COVID-19.

## Case presentation

Case 1

A 59-year-old male presented to the hospital with fever and dyspnea for five days. He did not have a history of T2DM or other comorbidities. He had a BMI of 23. He tested positive for COVID-19 on reverse-transcription polymerase chain reaction (RT-PCR) and was hospitalized with the diagnosis of hypoxemic respiratory failure. Blood gas analysis (BGA) was within normal limits, and he had a blood glucose level of 114 mg/dL. He did not receive steroids on admission since his oxygen saturation and BGA were normal.

On admission, the patient's vital signs were normal and his physical examination was unremarkable. He received supportive care and his hypoxemia showed significant improvement. However, on day three, he became lethargic, tachycardic, and tachypneic with an oxygen saturation of 95% on room air. Blood tests revealed an elevated anion gap metabolic acidosis a with pH of 7.3 (normal range: 7.35-7.45), bicarbonate of 10 mmol/L (normal range: 22-28 mmol/L), β-hydroxybutyrate of 5.4 mmol/L (normal level: <0.5 mmol/L), and glucose of 679 mg/dL (normal range: 70-110 mg/dL) (Table 1). He received treatment with continuous insulin infusion, electrolyte replacement, and massive intravenous hydration in the ICU. Once his DKA resolved, he was transitioned to a basal-bolus insulin regimen, and he remained insulin-dependent at the five-month outpatient follow-up.

Case 2

A 49-year-old male was transferred to our hospital due to acute respiratory failure and a positive test for SARS-CoV-2. He had no comorbidities or any familial history of diabetes mellitus. His preadmission HbA1c was 4.5% and he had a BMI of 24. His vital signs on admission were as follows: heart rate: 90 bpm, RR: 22 rpm, and oxygen saturation: 95% on room air. He received 5 liters of supplementary oxygen via nasal cannula with apparent improvement. On day two of hospitalization, the patient developed confusion, tachycardia, tachypnea, and his oxygen saturation was 90% on 5 liters of oxygen via nasal cannula. Laboratory tests showed abnormally elevated glucose of 625 mg/dL. BGA revealed a pH of 7.1, bicarbonate of 8 mmol/L, and β-hydroxybutyrate of 5 mmol/L; he was subsequently diagnosed with DKA (Table 1).

The patient was transferred to the ICU for further management. He was started on fluid resuscitation with isotonic normal saline, intravenous insulin infusion, antibiotics, and electrolyte replacement. After the resolution of his DKA, subcutaneous insulin was started. He was found to be insulin-dependent six months after his discharge.

Case 3

A 33-year-old male with normal glucose levels prior to admission was transferred to our hospital from an outpatient office with a two-day history of dyspnea and altered sensorium. He was tachycardic and tachypneic with an oxygen saturation of 96% on a 3L nasal cannula. He tested positive for COVID-19. His BMI was 25.5, and he had no other risk factors for diabetes and no family members with a history of diabetes. Physical examination showed no abnormalities except for the presence of rales on lung auscultation and marked accessory muscle use.

Severe DKA was diagnosed with a glucose level of 690 mg/dL, bicarbonate of 4 mmol/L, serum β-hydroxybutyrate of 5.8 mmol/L, and pH of 6.6. He was resuscitated with intravenous fluids and insulin infusion was started. DKA resolved after five days and he was discharged home on subcutaneous insulin. He remained insulin-dependent at the six-month follow-up.

Table 1 presents a summary of the laboratory findings of the three patients.

**Table 1:** Laboratory findings of the three patients with DKA and COVID-19 DKA: diabetic ketoacidosis; COVID-19: coronavirus disease 2019; BMI: body mass index

Variables	Normal value	Patient 1	Patient 2	Patient 3
pH	7.35–7.45	7.3	7.1	6.59
pCO_2_ (mmHg)	35–45	19.3	15.4	10.6
HCO_3_ (mmol/L)	22–28	10	8	4
Glucose (mmol/L)	3.9–5.5 (80-100 mg/dL)	38.9 (679 mg/dL)	35 (625 mg/dL)	40 (690 mg/dL)
Osmolarity (mOsm/L)	275–295	316	292	270.7
Serum beta-hydroxybutyrate (mmol/L)	0.4–0.5	5.4	5	5.8
BMI	18–29.9	23	24	25.5

## Discussion

We discussed the cases of three patients with SARS-CoV-2 infection complicated by new-onset diabetes mellitus and DKA. The SARS-CoV-2 infection was confirmed by RT-PCR. One patient presented with mild, one with moderate, and one with severe DKA, as summarized in Table 1. None of the patients had a family history of diabetes; fasting glucose and HbA1c were normal in all of them prior to being hospitalized.

Several studies indicate that SARS-CoV-2 infection can lead to new-onset diabetes mellitus, as well as acute hyperglycemic crisis such as DKA or hyperglycemic hyperosmolar state (HHS) in diabetic and non-diabetic patients, unmasking pre-existing diabetes or exacerbating the condition [11-13].

In their study, Li et al. reported that 42 (6.4%) out of 658 patients with SARS-CoV-2 manifested ketosis, while three of these patients developed DKA. Ketosis in patients with COVID-19 can prolong the length of hospital stay and increase the chances of mortality. DKA is an acute life-threatening complication of diabetes mellitus that requires early recognition in order to avoid fatal outcomes. It has been hypothesized that viral infections can lead to ketosis and DKA even in patients without a history of diabetes mellitus (13,14). Viral infections such as H1N1 and SARS-CoV-2 can also lead to ketosis-prone diabetes (KPD), a rare subtype of diabetes that presents with features of both type 1 and type 2 diabetes and is usually associated with DKA [14]. Wander et al., in a retrospective cohort study involving veterans, reported that patients with recent SARS-CoV-2 infection were at an increased risk of diabetes [15].

The mechanism by which SARS-CoV-2 triggers DKA in diabetic and non-diabetic patients may entail the secretion of counterregulatory hormones in response to the stress induced by infection and the direct viral damage to pancreatic islet cells, which leads to impaired insulin secretion. Furthermore, the pro-inflammatory state can accelerate lipolysis and induce the production of ketones, leading to ketosis and subsequent ketoacidosis [16].

There is a well-recognized relationship between diabetes mellitus and COVID-19. Many studies have reported severe cases and worse outcomes in patients with diabetes mellitus who developed COVID-19. However, recent studies have shown that SARS-CoV-2 infection can lead to persistent pancreatic damage that results in new-onset diabetes mellitus even in previously healthy individuals [16,17].

Several mechanisms have been proposed to explain the potential diabetogenic effect of SARS-CoV-2. There is evidence to suggest that viral infection can lead to the direct damage of pancreatic beta cells, which in turn can result in defective insulin secretion [16,18]. However, the expression of angiotensin-converting enzyme 2 (ACE-2) in human pancreatic tissue is not clearly defined, but it is known that ACE-2 and transmembrane serine protease 2 (TMPRSS-2) are highly expressed in exocrine pancreas microvasculature and ductal pancreatic tissue [19]. The viral infection activates a type-1 immune response that results in the production of TNF, IFN-gamma, and IL-6, which induce transient insulin resistance in muscle tissue and the liver. In response to this state of insulin resistance, the pancreas increase insulin secretion, which promotes an antiviral immune response [18]. Furthermore, viral infection can lead to oxidative stress that results in hypoxia and inflammation that impairs glucose homeostasis [19]. On the other hand, SARS-CoV-2 infection leads to ACE-2 downregulation and subsequent overactivation of the renin-angiotensin-aldosterone system (RAAS), which leads to increased levels of angiotensin II, which can also induce insulin resistance and increase hepatic glucose production [16,20]. Nevertheless, further research is needed on the specific mechanisms of pancreatic damage.

Some specific considerations must be taken related to the management of patients presenting with DKA in the background of COVID-19. The traditional management of acute hyperglycemic complications such as DKA includes an intravenous insulin regimen in the hospital setting. However, due to the high contagiousness of the virus and in order to limit healthcare workers' exposure to the virus, ambulatory home management of DKA with subcutaneous insulin has been proposed as a viable option for mild to moderate cases. Furthermore, SGLT-2 inhibitors must be used with caution in these patients because they have the potential to trigger DKA [12,16].

Shrestha et al., in a meta-analysis, showed that 19.70% of COVID-19 patients also have diabetes. Also, patients with new-onset diabetes after infection with COVID-19 have higher mortality rates and worse outcomes when compared to non-diabetic patients [20]. However, to date, there have been few reports of patients in whom diabetes persists after the initial presentation. Further studies need to be conducted among patients with persistent diabetes after SARS-CoV-2 infection to gain deeper insights into the topic.

## Conclusions

COVID-19 may cause metabolic complications resulting in DKA even in patients without a history of diabetes mellitus. New-onset diabetes after SARS-CoV-2 infection is being increasingly recognized. There is increasing evidence that SARS-CoV-2 infection can lead to permanent pancreatic damage, resulting in diabetes mellitus. However, more studies need to be conducted to better understand the mechanism behind it.
